# Supplementary material for: The interplay of demography and selection during maize domestication and expansion
Source: Genome Biol. 2017 Nov 13;18:215. doi: 10.1186/s13059-017-1346-4 (PMC5683586; doi:10.1186/s13059-017-1346-4)
Supplement: Supplementary file 1 — Figure S1. Demography of maize populations. A. MSMC results before and after masking candidate regions under selection during domestication. B. Percentage of heterozygous sites versus distance from the Balsas Valley in 3520 samples from the SeeDs data set. Figure S2. Boxplot of multiple population genetic statistics. Watterson’s theta (A), θ π (B), and Tajima’s D (C) are based on values in 10-kb non-overlapping windows across the genome. Percentage of derived homozygous sites was calculated for each individual and reported per population (D). Figure S3. Cumulative length of ROHs in cM across populations. Lines indicate median values in each population. ROH runs of homozygosity. Figure S4. Calculation of D statistic across populations. Evidence of introgression from mexicana into Mexican highland, Guatemalan highland, and southwestern US highland maize populations. The dashed lines correspond to Z-scores equal to −10 and 10. Figure S5. $\hat {f_{d}}$fd^ statistic results. Loess regression of $\hat {f_{d}}$fd^ in 10-kb nonoverlapping windows across all chromosomes. Figure S6. Relative burden of deleterious alleles under additive model between maize and teosinte (A; mean value in teosinte population was used as the standard to calculate the relative burden) and among maize populations (B; mean value in Mexican lowland population was utilized as the standard to calculate the relative burden). Figure S7. Relative burden of deleterious alleles under both additive and recessive models with differen [file 13059_2017_1346_MOESM1_ESM.pdf]

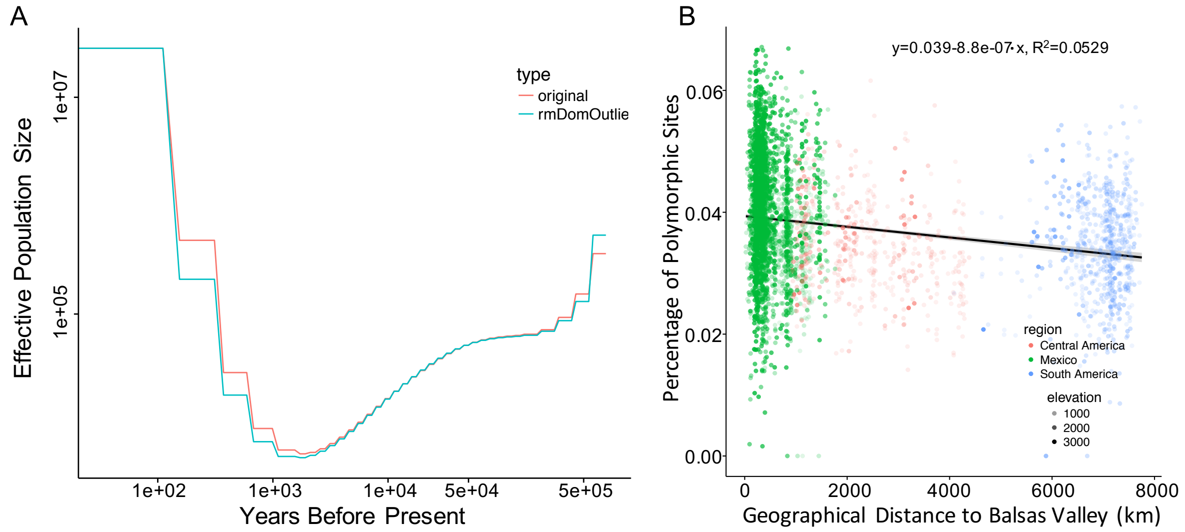

Figure S1. Demography of maize populations. A. MSMC results before and after masking candidate regions under selection during domestication. B. Percentage of heterozygous sites versus distance from the Balsas Valley in 3520 samples from the SeeDs data set.

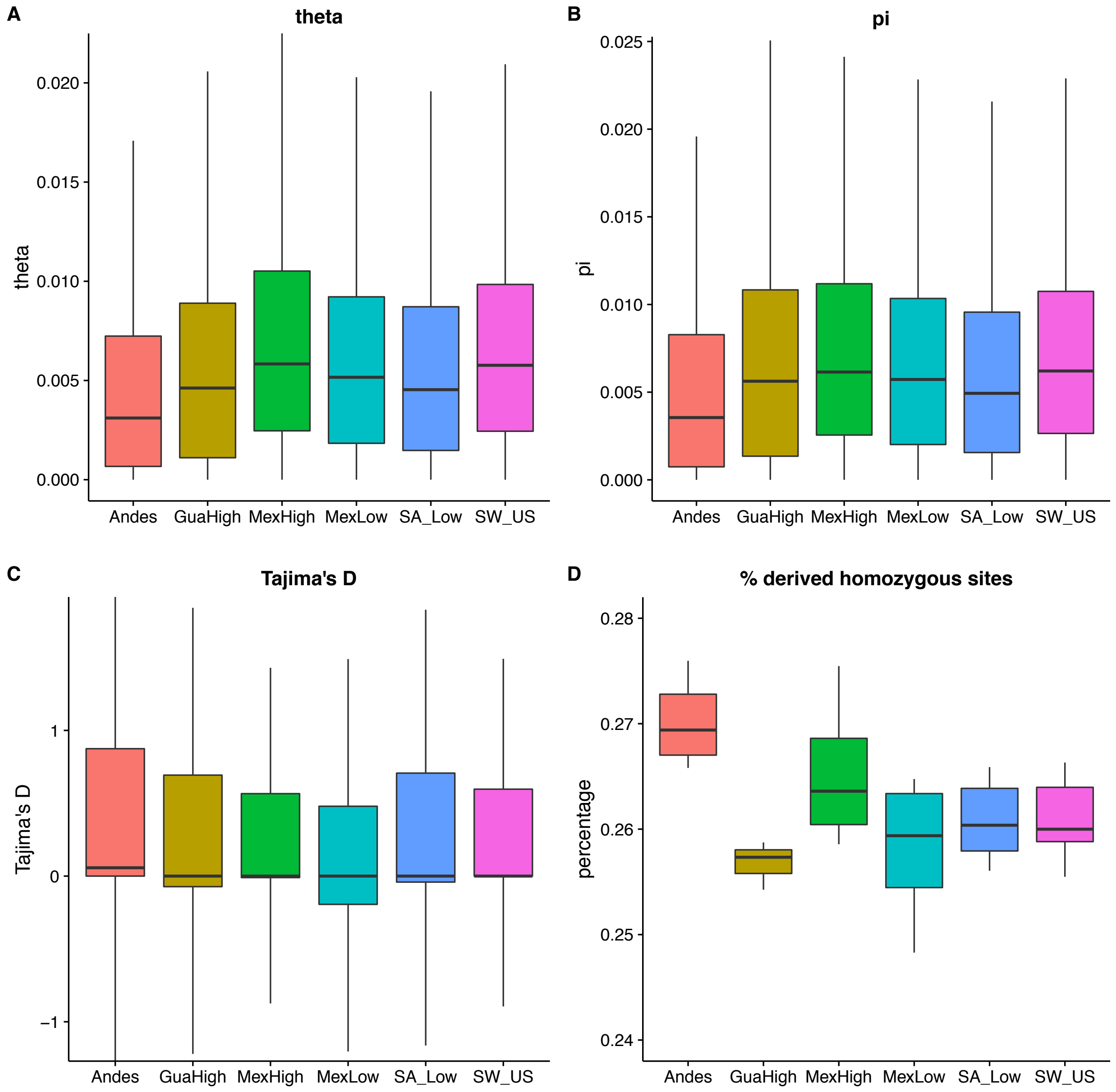

Figure S2. Watterson's theta (A), θ_{π} (B) and Tajima's D (C) are based on values in 10-kb non-overlapping windows across the genome. Percentage of derived homozygous sites was calculated for each individual and reported per population.

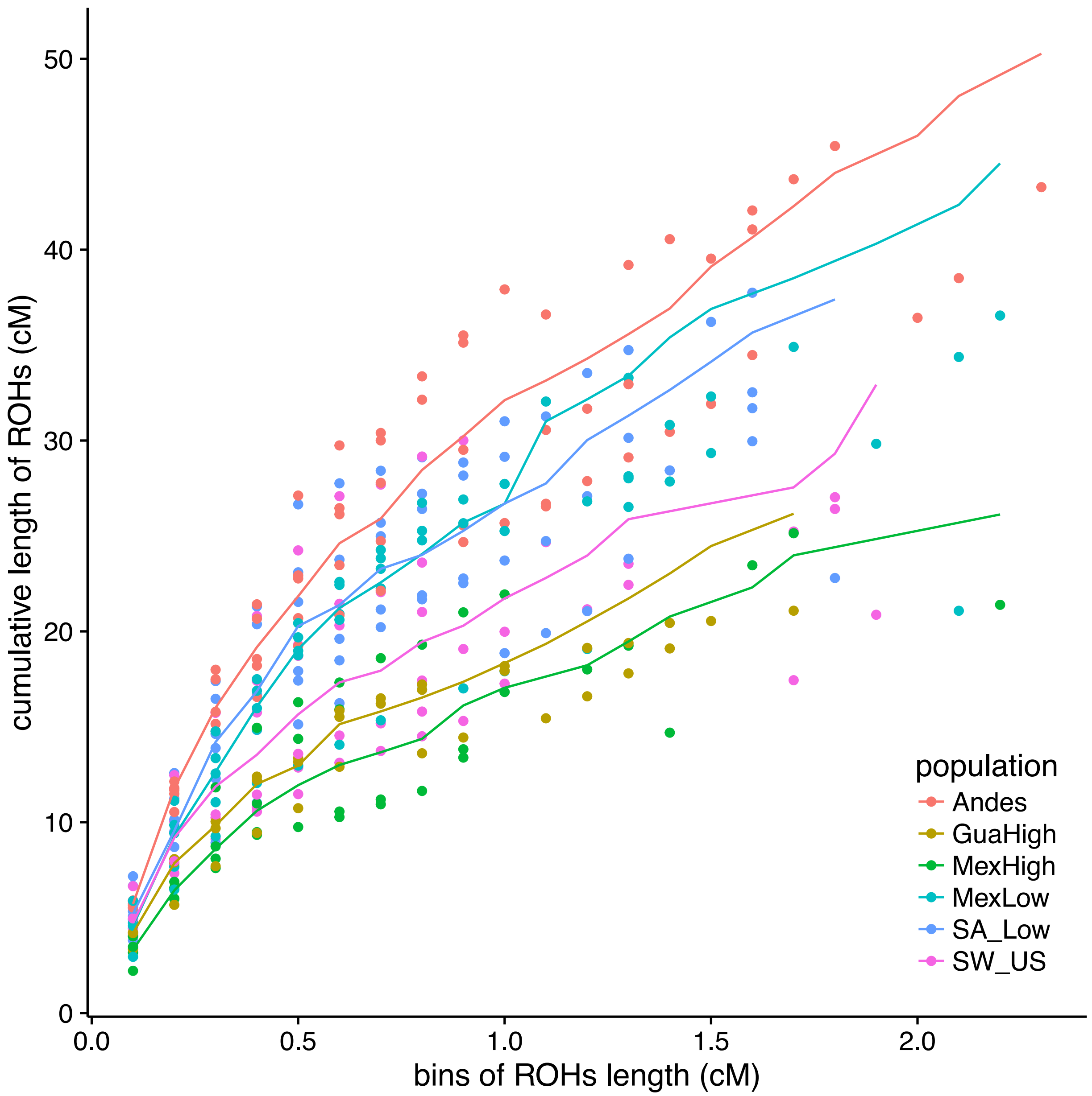

Figure S3. Cumulative length of ROHs in cM across populations. Lines indicate median values in each population. ROH: runs of homozygosity.

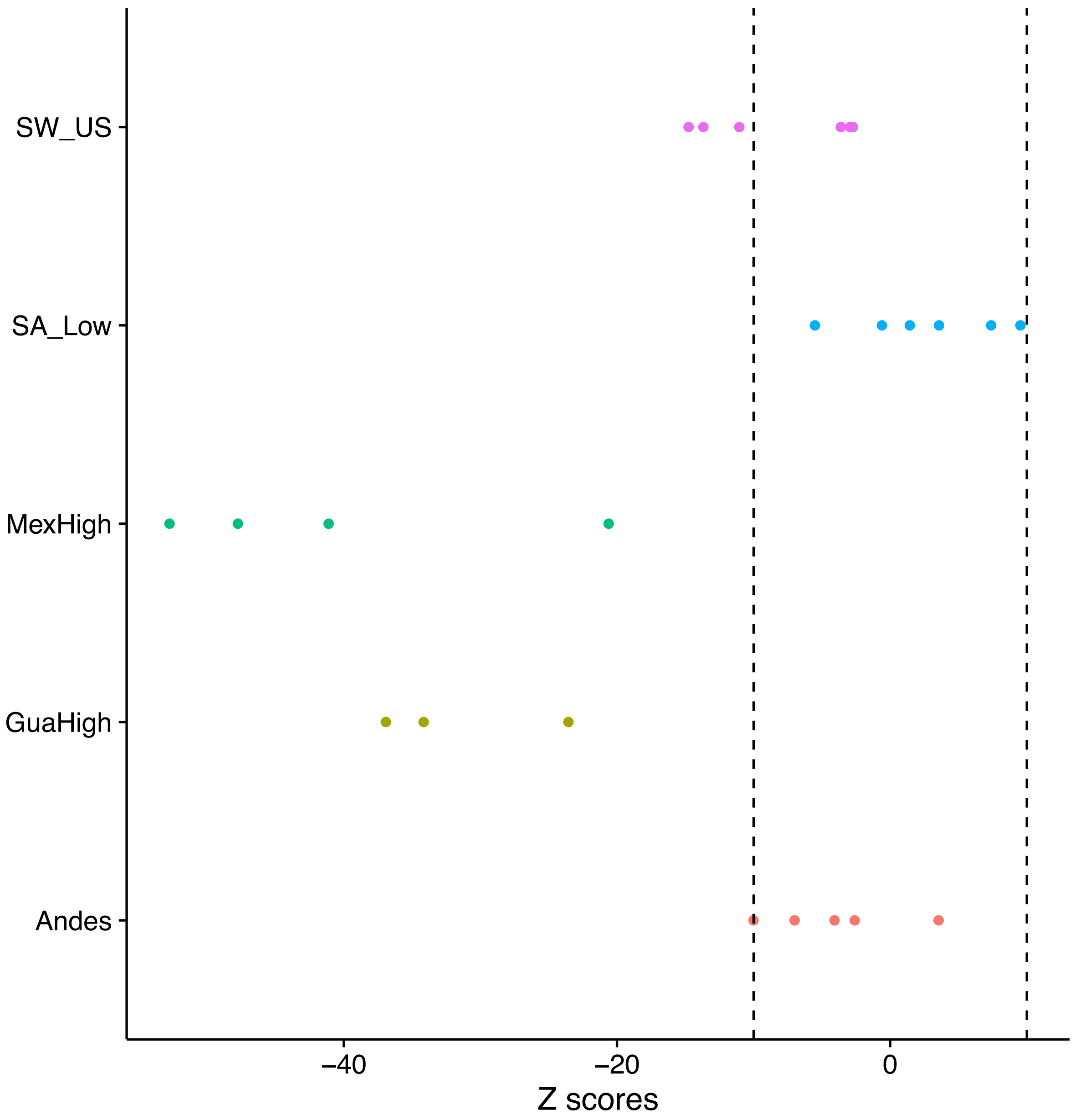

Figure S4. Calculation of D statistic across populations. Evidence of introgression from *mexicana* into Mexican highland, Guatemalan highland and Southwestern US highland maize populations. The dashed lines correspond to Z scores equal to -10 and 10.

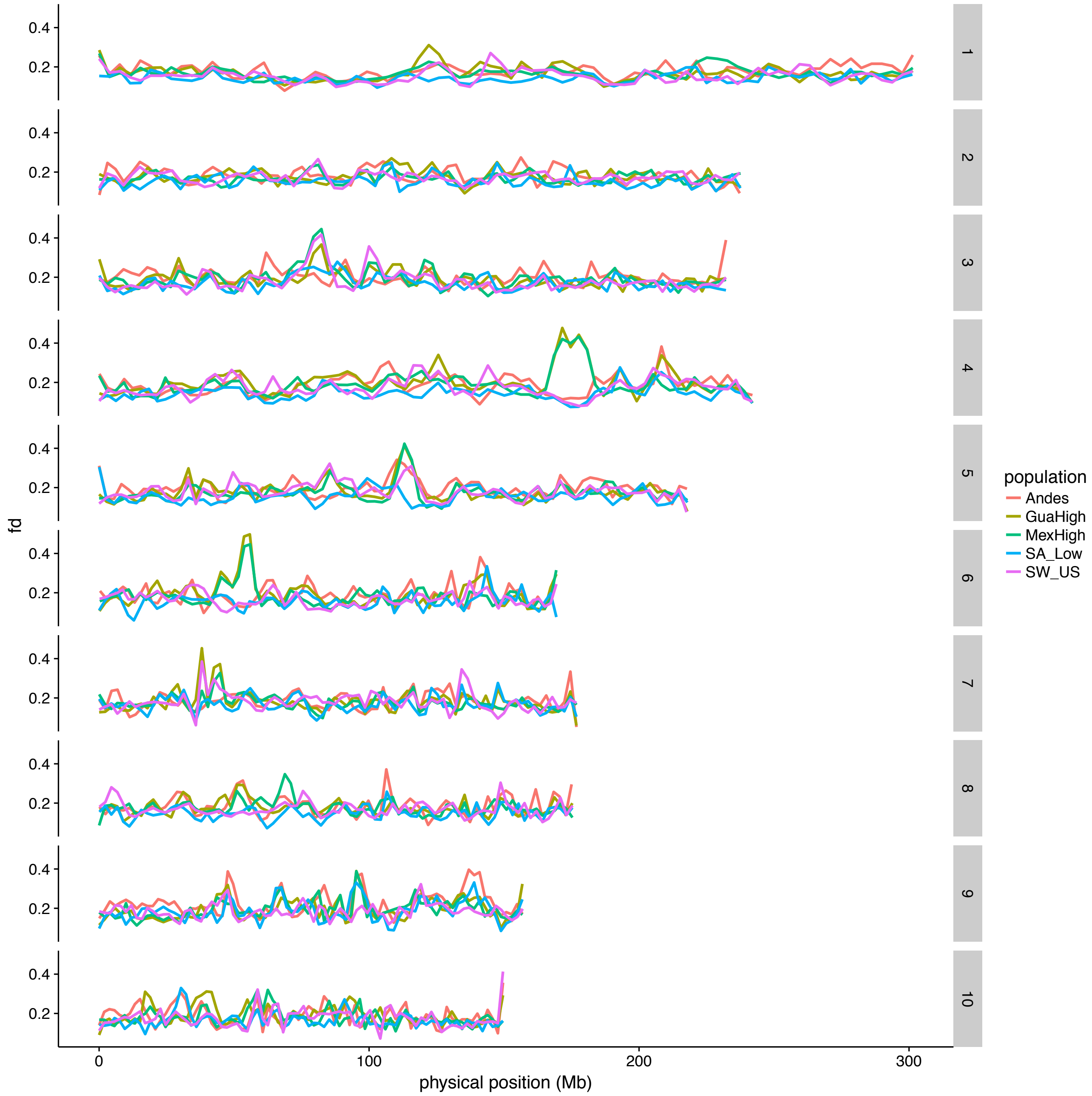

Figure S5. f_d statistic results. Loess regression of f_d in 10-kb non-overlapping windows across all chromosomes.

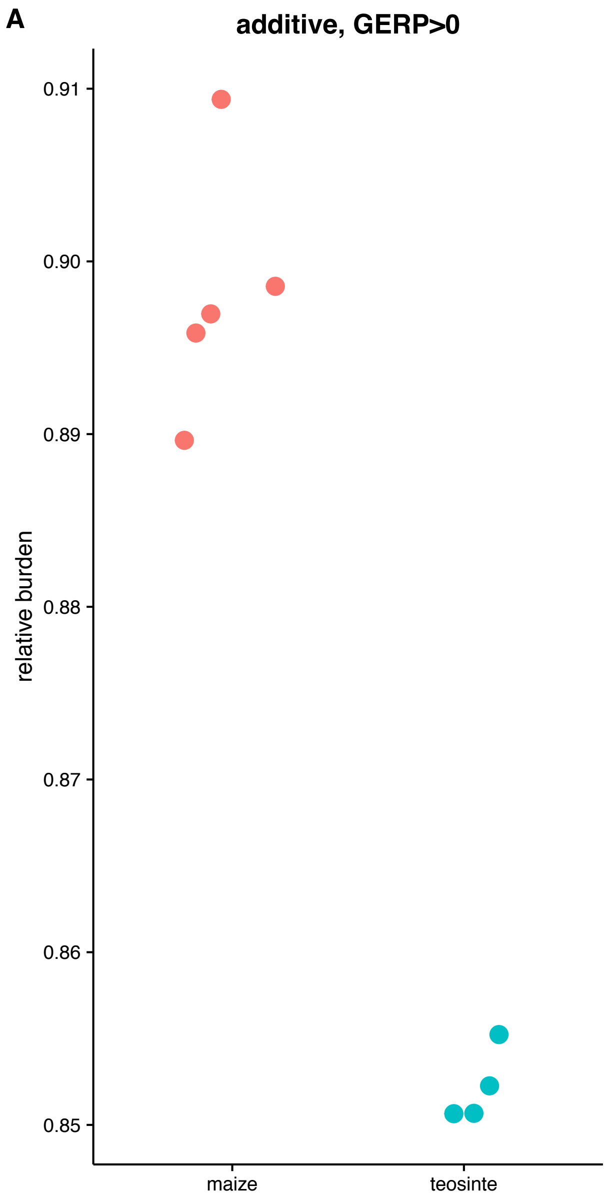

Figure S6. Relative burden of deleterious alleles under additive model between maize and teosinte (A) and among maize populations (B).

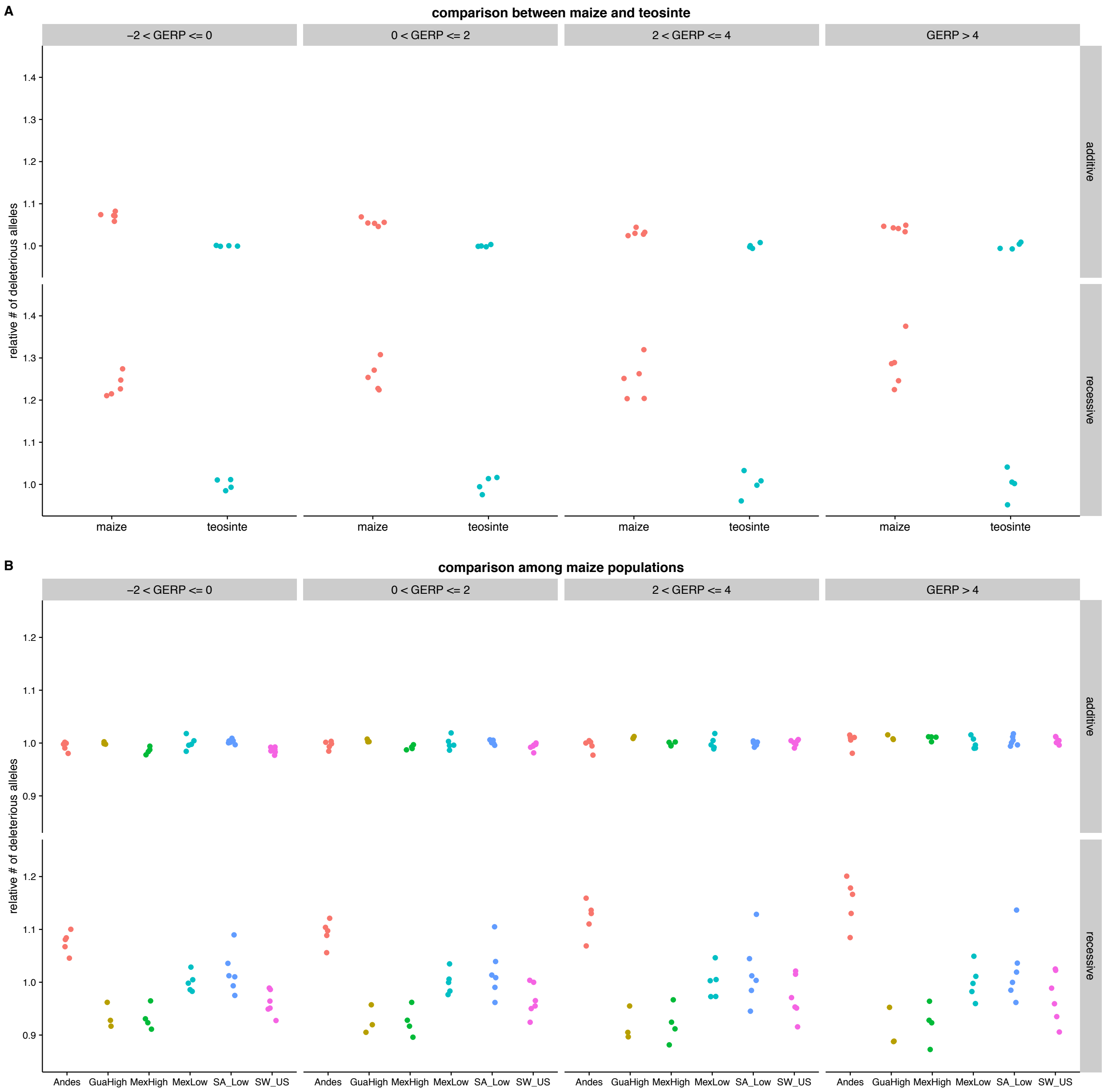

Figure S7. Relative burden of deleterious alleles under both additive and recessive models with different GERP partitions between maize and teosinte (A) and among maize populations (B).

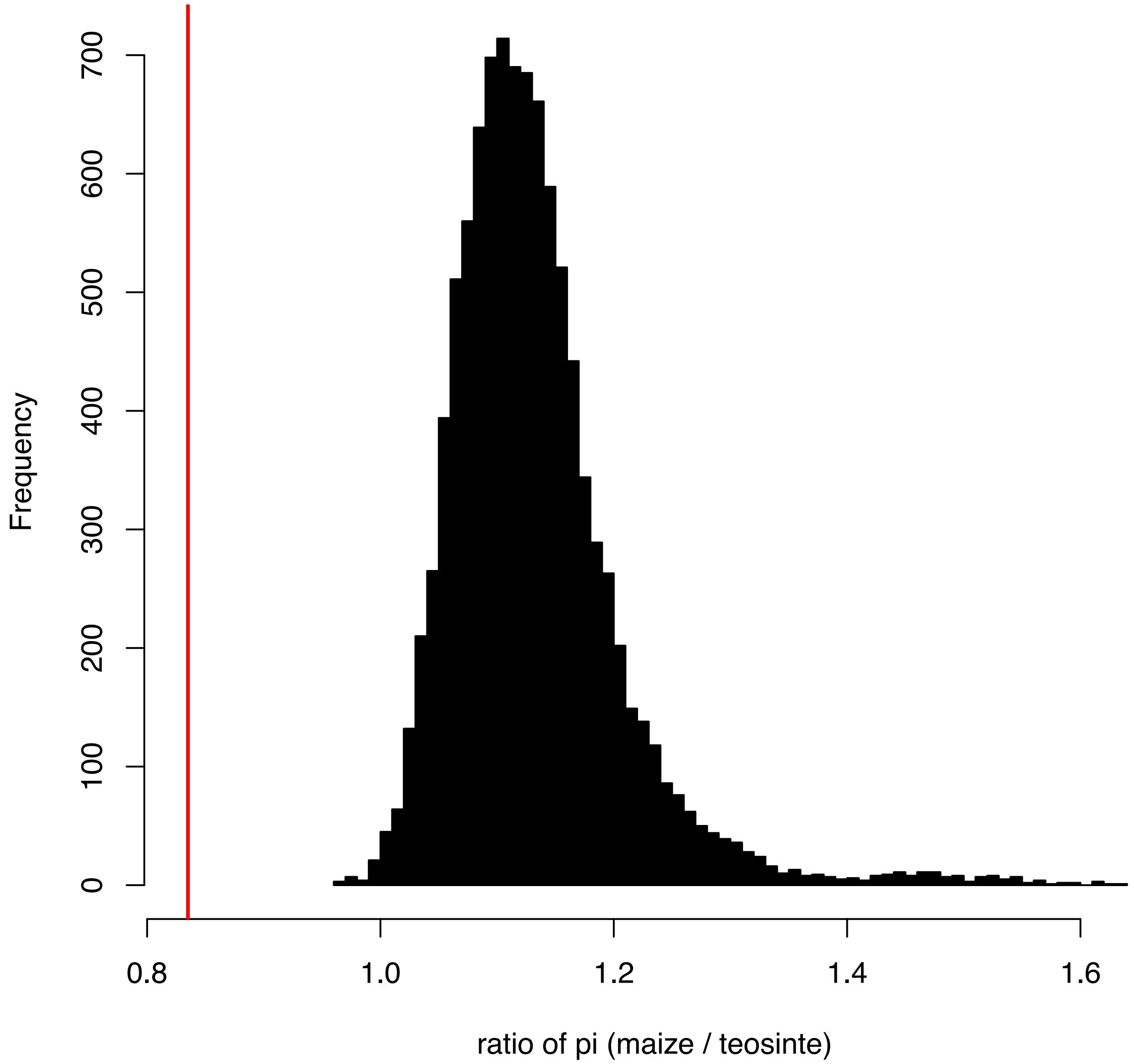

Figure S8. Domestication candidate genes exhibited lower θ_π ratio between maize and teosinte, a signal of selection in these genes. Distribution of ratio of θ_π between maize and teosinte in 420 domestication candidate genes (mean value was indicated with red line) against 10,000 replicates of genome-wide sampling of 420 random genes.

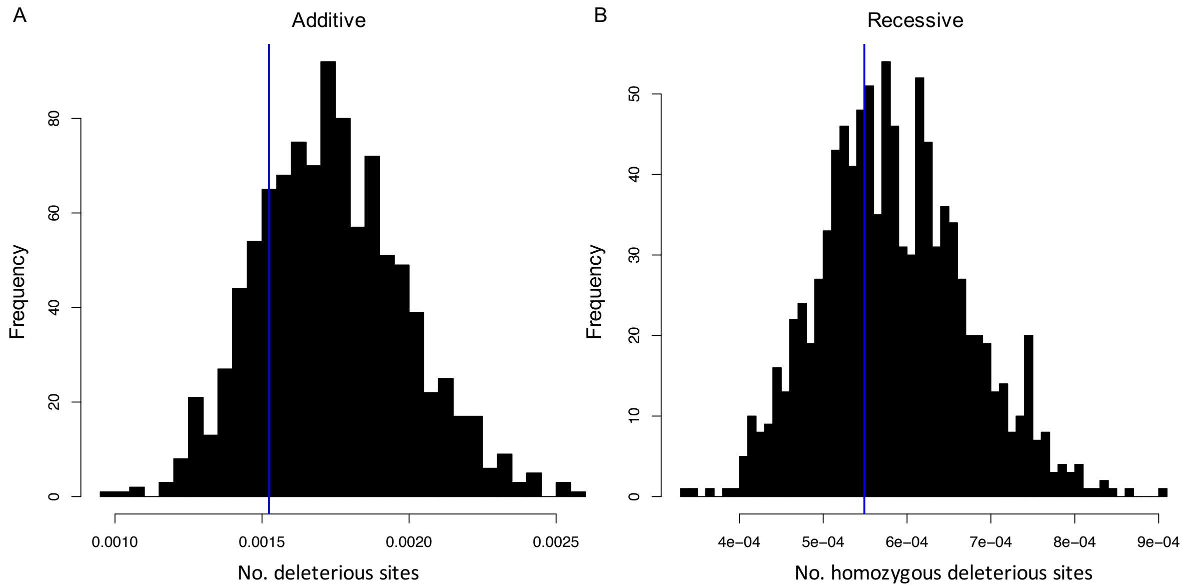

Figure S9. Distribution of number of deleterious sites per bp in 420 domestication candidate genes (indicated with blue line) compared to genome-wide random samples under an (A) additive model and (B) recessive model.

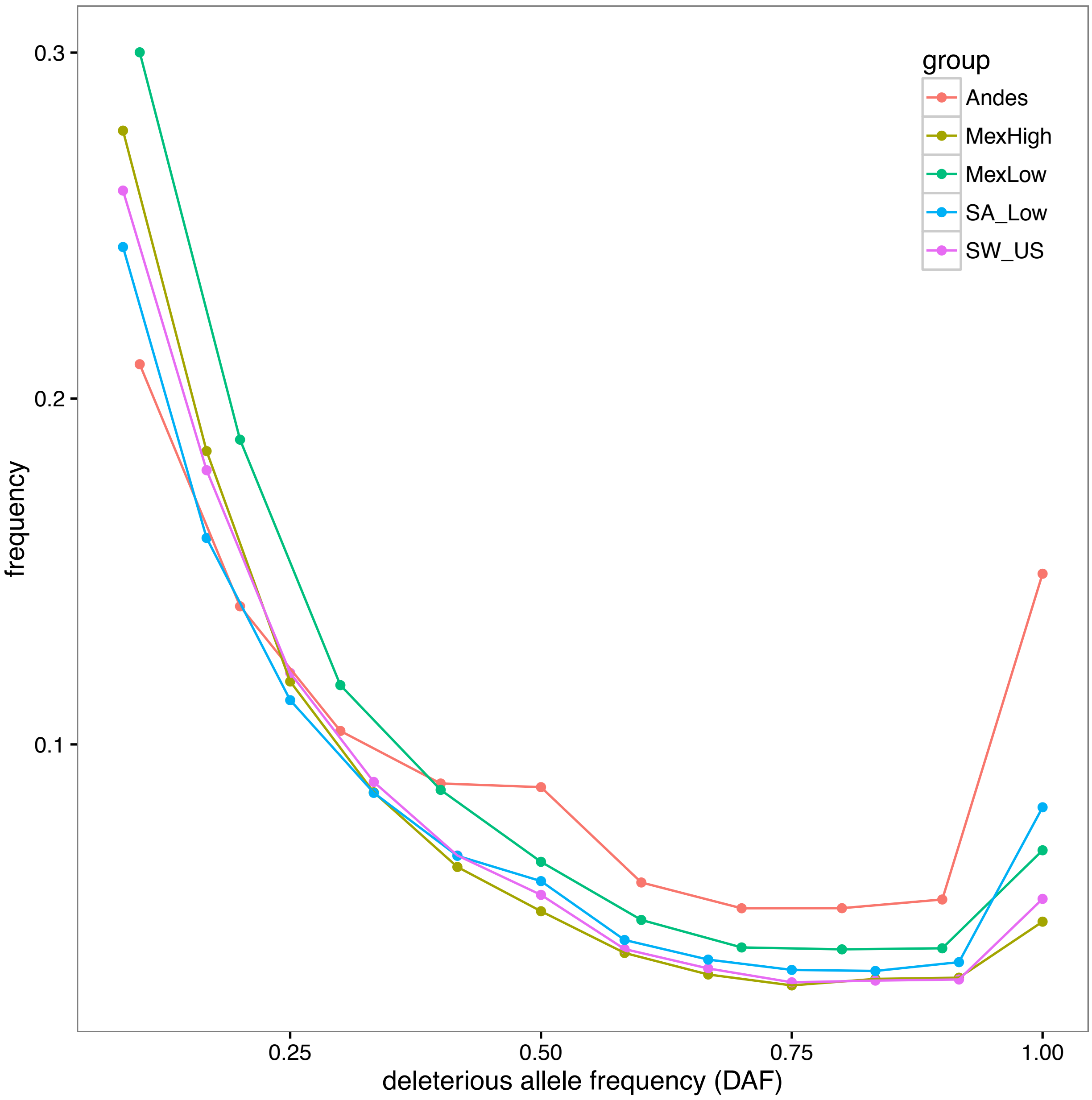

Figure S10. Site frequency spectrum of deleterious SNPs in five populations. GuaHigh is not included since the small sampling limited power for the SFS.

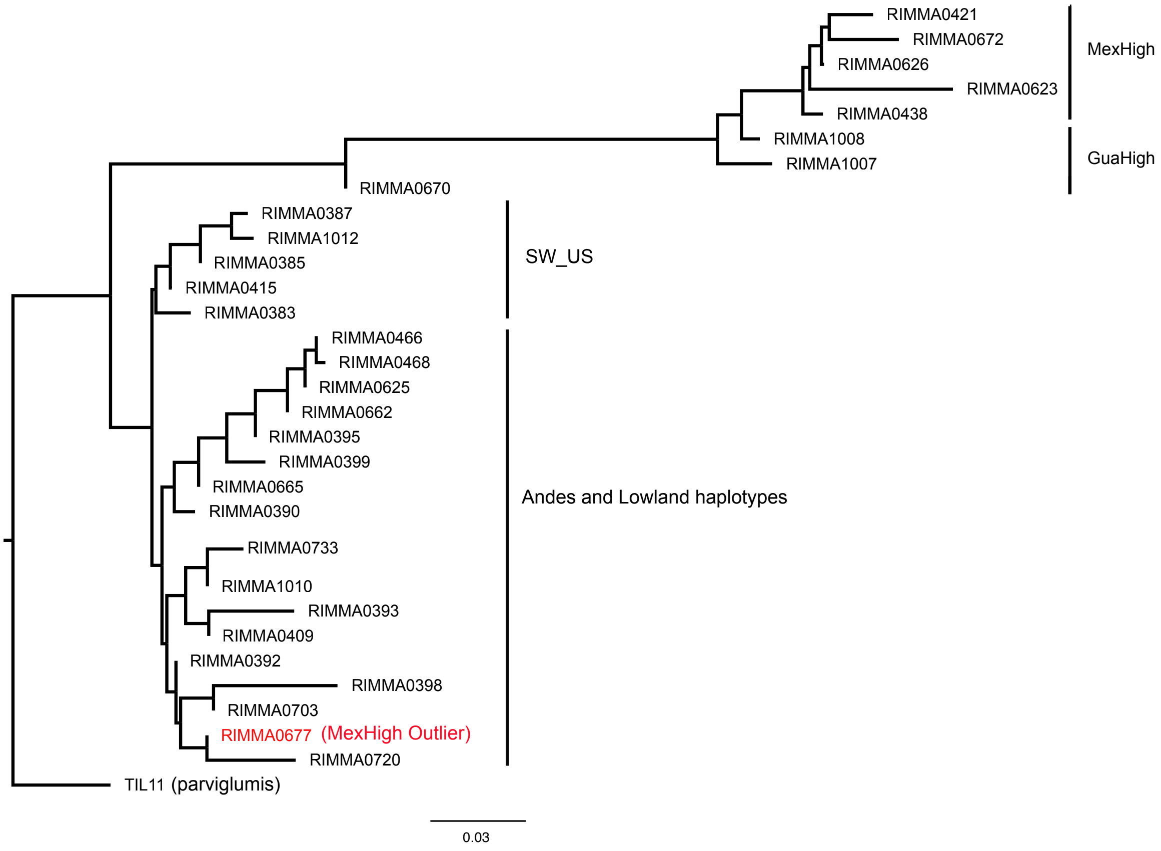

Figure S11. Neighbor Joining tree of SNPs from an inversion on chromosome 4 with a diagnostic haplotype for highland Mexican material.
